# Cheoplastic Process of Mounting Teeth

**Published:** 1857-04

**Authors:** 


*Cheoplastic Process of Mounting Teeth.*—The above method of mounting artificial teeth, is attracting considerable attention, and has already been adopted by many practitioners.

We published in the preceding number of the Journal, a letter from Professor Austen, in answer to some inquiries concerning it, which the senior editor addressed to him, knowing that he had had considerable experience in mounting teeth in this way. But hitherto we have refrained from expressing our opinion with regard to its comparative merits. Indeed, we determined not to do so, until we had had the fullest opportunity of judging of its practical value and advantages.

Of these we are now so thoroughly convinced, that we have no hesitation in saying, that this method of mounting artificial teeth, secures, when done with proper care and skill, greater accuracy of adaptation of the base to the model, and more comfort and usefulness to the patient, than by any other method heretofore practiced.

The process of mounting is also attended with much less labor and expense.

We hope to be furnished, soon, with a description of it for publication in the Journal.

Dr. Dwinelle, in reply to certain inquiries of the senior editor with regard to this new process, expresses his opinion concerning it as follows:

*No.* 3 *Bond Street, New York, March* 30, 1857.

Dr. C. A. Harris,

*Dear Sir:* In reply to your letter of the 26th inst., inquiring whether I have adopted the cheoplastic process of mounting artificial teeth in my practice, and if so, what my opinion is as to its advantages, if any, over the methods heretofore in use. I take great pleasure in responding that, after fully testing it, I unhesitatingly accord to it the following advantages:

First.—The great beauty, simplicity and perfection of the detail of the process which secures to you the closest approximation to the end desired, at every stage of your labor.

Second.—Its great facility and the necessarily perfect adaptation to the impression and consequent absolute accuracy to fit.

Third.—Its application under all circumstances.

Fourth.—Its non-corrosiveness, being equal to gold or platina in this respect. Its tastelessness and cleanliness, together with the remarkable ease and comfort with which it is worn by the patient.

Fifth.—The unusual facility which it gives the operator to restore the mouth and face to their original form, the work of hours by any of the old processes is now accomplished in a few moments.

Sixth.—The ease and safety with which it can be repaired.

Seventh.—Its advantage in requiring no additional apparatus in the laboratory of the dentist, for its construction.

Lastly, in point of economy and expense, there is no process known in our profession equal to it.

Dr. Blandy deserves great credit for his untiring labor in bringing about a result which our whole profession must sooner or later, endorse as one of its most valuable improvements of the age.

Very truly, your friend, WM. H. DWINELLE.

